# Evaluating the impact of a quality management intervention on post-abortion contraceptive uptake in private sector clinics in western Kenya: a pre- and post-intervention study

**DOI:** 10.1186/s12978-018-0452-4

**Published:** 2018-01-19

**Authors:** Susy Wendot, Rachel H. Scott, Inviolata Nafula, Isaac Theuri, Edward Ikiugu, Katharine Footman

**Affiliations:** 1 Marie Stopes Kenya, Kindaruma Road, P.O. Box 59328-00200, Nairobi, Kenya; 2 UCSF-Global programs, Morning side office park, Ngong Road, P.O Box 40821-00100, Nairobi, Kenya; 3 Marie Stopes International, Conway Street, London, W1T 6LP UK; 4 Department of Population Health, London School of Hygiene and Tropical Medicine, Keppel Street, London, WC1E 7HT UK

**Keywords:** Post-abortion contraception, Post-abortion family planning, Contraception, Family planning, Abortion, Comprehensive abortion care, Quality of care

## Abstract

**Background:**

Integration of family planning counselling and method provision into safe abortion services is a key component of quality abortion care. Numerous barriers to post-abortion family planning (PAFP) uptake exist. This study aimed to evaluate the effect of a quality management intervention for providers on PAFP uptake.

**Methods:**

We conducted a pre- and post-intervention study between November 2015 and July 2016 in nine private clinics in Western Kenya. We collected baseline and post-intervention data using in-person interviews on the day of procedure, and follow-up telephone interviews to measure contraceptive uptake in the 2 weeks following abortion. We also conducted semi-structured interviews with providers. The intervention comprised a 1-day orientation, a counselling job-aide, and enhanced supervision visits. The primary outcome was the proportion of clients receiving any method of PAFP (excluding condoms) within 14 days of obtaining an abortion. Secondary outcomes were the proportion of clients receiving PAFP counselling, and the proportion of clients receiving long-acting reversible contraception (LARC) within 14 days of the service. We used chi-squared tests and multivariate logistic regression to determine whether there were significant differences between baseline and post-intervention, adjusting for potential confounding factors and clustering at the clinic level.

**Results:**

Interviews were completed with 769 women, and 54% (414 women) completed a follow-up telephone interview. Reported quality of counselling and satisfaction with services increased between baseline and post-intervention. Same-day uptake of PAFP was higher at post-intervention compared to baseline (aOR 1.94, *p* < 0.001), as was same-day uptake of LARC (aOR 1.72, *p* < 0.001). There was no overall increase in uptake of PAFP 2 weeks following abortion. Providers reported mixed opinions about the effectiveness of the intervention but most reported that the supervision visits helped them improve the quality of their services.

**Conclusions:**

A quality management intervention was successful in improving the quality of PAFP counselling and provision. Uptake of same-day PAFP, including LARC, increased, but there was no increase in overall uptake of PAFP 2 weeks after the abortion.

## Plain English summary

Approximately 40% of pregnancies worldwide are unintended, because of ineffective or non-use of contraception, or method failure, and around half of these pregnancies end in abortion. Access to safe abortion care is limited in Kenya, and many women suffer morbidity or mortality from unsafe abortions. Post-abortion family planning (PAFP) as part of safe abortion care increases contraceptive prevalence and reduces unintended pregnancies and hence unsafe abortion. Reducing provider barriers to counselling on family planning could be important in improving PAFP uptake. This study aimed to evaluate the effect of a quality management intervention on PAFP uptake. The intervention targeted providers and comprised a 1-day training session, a counselling job-aide, and supportive supervision visits. Women reported higher satisfaction with the service after the intervention compared to before, with more reporting that the provider spent enough time on the consultation, asked them about previous methods used and whether these methods suited them, and gave clear instructions. Women were more likely to take PAFP on the day of the abortion, including long acting contraceptive methods, after the intervention compared to before, but there was no difference in the proportion taking any PAFP within 2 weeks of the abortion. In interviews, providers said that the supervision visits helped them to improve the service they provided. The intervention may have support providers to help women decide and take a contraceptive method more quickly, i.e. on the day, but does not appear to have affected women’s likelihood of taking up any method of PAFP within 2 weeks of the procedure.

## Background

Approximately 40% of pregnancies worldwide are unintended, because of ineffective or non-use of contraception, or method failure. Estimates suggest that half of these unintended pregnancies result in induced abortions [1]. In Kenya, an estimated 464,000 induced abortions occurred in 2012; an abortion rate of 48 per 1000 women [2]. In 2010 the law was changed so that abortion may be granted to a pregnant woman or girl when, in the opinion of a trained health professional, she needs emergency treatment or her life or health is in danger [3]. However, access to safe and high quality abortion care is limited in Kenya, and an estimated 120,000 women were treated for complications from unsafe abortion in 2012 [2].

Integration of family planning (FP) counselling and method provision into safe abortion services is a key component of quality abortion care [4]. Post-abortion family planning (PAFP) increases contraceptive prevalence and reduces unsafe abortion and associated maternal mortality and social costs [5]. However, PAFP uptake is low in Kenya [6]. Numerous barriers to PAFP exist, including facility-level barriers such as a lack of contraceptive methods and trained staff, provider-level barriers such as lack of knowledge and denial of methods to certain groups, and client-level barriers such as fear of side effects and partner disapproval [5]. Although evidence suggests that counselling women on FP following an abortion can reduce client-level barriers to PAFP in low-income countries [7], little research has assessed approaches to remove provider-level barriers to effective counselling on PAFP. Quality of care for abortion in private clinics in Kenya is often low, with limited provision of FP options to abortion clients [8], and mechanisms to improve quality of care, including PAFP, are needed.

This study aimed to evaluate the effect of a quality management intervention on PAFP uptake. Quality management aims to identify opportunities and implement measures to improve quality of care [9]. In abortion care, a quality management intervention might consider equipment and infrastructure; staffing and staff training; record keeping; or processes, among other factors, in order to improve quality of care [9]. In this case, the quality management intervention (described in detail below) was targeted at providers and aimed to reduce provider barriers to high quality counselling on PAFP. The objectives of this study were (1) to assess whether PAFP and long-acting reversible contraceptive (LARC) uptake increased after the introduction of a quality management intervention in private clinics in Western Kenya and (2) to understand the drivers and barriers experienced by private providers regarding their provision of PAFP.

## Methods

To assess the effect of a quality management intervention on PAFP uptake, we conducted a pre- and post-intervention study between November 2015 and July 2016 in Marie Stopes Kenya’s (MSK) social franchise network in Western Kenya. The study took place in private sector clinics that are part of a social franchise network, which means they have been branded and trained by MSK, and receive ongoing quality assurance and support with demand generation and product supply. Clinics were selected based on the following criteria: (1) minimum monthly case load of 12 safe abortion/post-abortion care (PAC) services per month in the past 3 months; (2) offer a range of FP methods; (3) have an updated memorandum of understanding with MSK for the year 2015; (4) not participating in any other study during the study period related to safe abortion. All 12 clinics in the Western Region meeting these criteria were invited to participate in the study and all accepted, but three clinics later dropped out, resulting in a total of nine clinics. Of the nine clinics, four were rural and five were urban. In all but one of the clinics the main provider was male. In seven of the clinics the main provider was a nurse, in one it was a doctor and in one it was a clinical officer. We collected baseline data from November 2015 to February 2016; the intervention started in February 2016; and we collected post-intervention data between March and July 2016. We conducted semi-structured interviews with providers in August 2016.

### Intervention

**Fig. 1:**
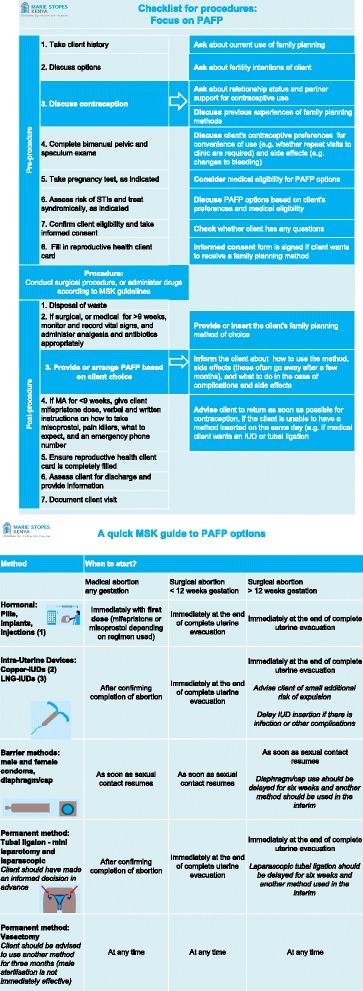
Study job aid given to providers

The intervention was a quality management intervention aiming to increase uptake of highly effective methods of contraception – that is to say, hormonal user dependent methods, long acting reversible contraceptive (LARC) methods, and permanent methods – following abortion. Condoms and traditional methods were not considered highly effective methods of contraception, though condoms were encouraged as dual protection against sexually transmitted infections. In addition to their lower effectiveness, condoms alone were not considered PAFP as providers may try to improve their PAFP provision by simply distributing condoms to post-abortion clients, rather than providing quality counselling on family planning. The intervention was made up of three components. First, providers attended a 1-day orientation on PAFP. The orientation included: (1) a discussion on the importance of PAFP provision; (2) re-orientation and role-play to practice balanced FP counselling; (3) training on a job aide given to the service providers; (4) values clarification; and (5) re-orientation on data reporting for PAFP. We reimbursed providers for their transportation and expenses. Secondly, a job aide consisting of a one-page guide to PAFP was given to the providers at the training. It included a checklist for safe abortion provision with a focus on PAFP counselling, and a description of when each contraceptive method can be provided post-abortion (Fig. 1). The final element of the intervention was enhanced supervision visits. All franchised service providers are visited once a month by a supervisor, to collect service data and provide supportive supervision. For the intervention, we developed a supervision checklist to ensure that all monthly supervision visits were structured to include a review of the provider’s PAFP provision, and a discussion of how the provider could improve contraceptive counselling and uptake. An action plan was included in each checklist, so that providers and supervisors could discuss action points to address any shortfalls in data quality, PAFP provision and supplies.

### Quantitative data collection and analysis

The primary outcome of the study was the proportion of clients receiving any method of PAFP (excluding condoms) within 14 days of receiving an abortion service from the study providers. Secondary outcomes were the proportion of clients receiving contraceptive counselling on the day of the service, and the proportion of clients receiving LARC within 14 days of the service. The study population was clients accessing safe abortion and PAC services at the selected clinics within the study period. For inclusion in the study, women had to be aged 18 years and above and have provided written informed consent.

The expected sample size for each stage of data collection (pre- and post-intervention) was 428 women. The sample size was designed to detect a 15-percentage point increase in PAFP from an expected baseline uptake of 45%, with 80% power and 95% confidence. The expected baseline uptake of 45% was drawn from routinely collected data by Marie Stopes Kenya on average PAFP uptake across all social franchise clinics. The sample size accounted for an expected 20% loss to follow up. At each stage of data collection (pre- and post-intervention), women were recruited from clinics until each had 36 participants, or until 3 months after the start of the data collection phase, based on an expected 50% response rate.

Trained research assistants collected data on socio-demographic status, counselling and provision of PAFP, fertility intentions, previous contraceptive use and satisfaction with the service received through in-person structured interviews with clients immediately after they had received the service. The research assistants collected information on contraceptive uptake through telephone-interviews 2 weeks later. Research assistants made two phone calls to study participants, and if the respondent did not answer either call then the woman was considered lost to follow up. The respondents were informed which day the call would be made, and research assistants checked with the respondent whether this would be a convenient day for the woman to receive a call, and if there was a preferred time for call back.

We double entered and cleaned the data using Epi Data. We used chi-squared tests to determine whether there were significant differences between pre- and post-intervention groups, and fitted a multi-level random effects logistic regression model, to adjust for differences between sample characteristics at baseline and post-intervention and potential confounding factors, and to take into account the hierarchical nature of the data, i.e. that individuals are clustered within clinics. We adjusted for age group, educational level, occupation, marital status, number of children, fertility intentions, type of abortion (medical or surgical), clinic, and whether the client was using contraception before the abortion. Data from the clinics that dropped out are not included in this analysis. We conducted all analyses using Stata 13.0.

### Qualitative data collection

Trained interviewers conducted semi-structured interviews with the nine providers in August 2016, using an interview guide to structure the interviews and gather information about the providers’ experience and perceptions of the intervention. We asked them whether they were willing to take part in an in-depth interview when we took their initial informed consent to take part in the study. Then we re-visited each clinic at the time we conducted interviews and asked whether they were still willing to take part. All nine providers agreed to take part. We used these interviews to understand the providers’ perceptions of abortion and PAFP provision, and barriers to client uptake of PAFP. We conducted interviews in English in the providers’ offices or consultation rooms. We audio recorded the interviews, upon consent of the providers, and recordings were then transcribed. We then single-coded the interviews and classified the emerging themes into broad themes to facilitate analysis.

## Results

### Quantitative

#### Sample size and response rates

Of 1653 women attending the clinic during the study period, 883 were approached for an interview (53%). In most instances, where women were not approached it was because the research assistant was not present. Response rates were high; interviews were completed for 769 (97%) of eligible women who were approached. Interviews were completed with 400 women at baseline and with 369 women post-intervention. Interview completion rates did not vary between baseline and post-intervention. There were some differences between client characteristics at baseline and post-intervention: women at post-intervention had a higher level of education, and were more likely to have had a surgical abortion (Table 1). Although 92% of women interviewed were willing to be followed up by phone, only 54% (414 women) completed a follow up interview. Follow up was higher at baseline (60%) than post-intervention (48%). Follow up interviews were completed with 239 women at baseline and 177 women post-intervention. Women lost to follow up were more likely to be young, to be studying, and to have no children than those who were followed up (results not shown). There was no difference in same-day uptake of PAFP between women who were followed up and those who were not.

**Table 1:** Client characteristics at baseline and post-intervention

	All	Baseline	Post-intervention	
	*N* (%)	*N* (%)	*N* (%)	*p*-value
Age group				0.309
18–24	427 (55.5)	213 (53.3)	214 (58.0)	
25–29	180 (23.4)	95 (23.8)	85 (23.0)	
30–34	84 (10.9)	47 (11.8)	37 (10.0)	
35+	78 (10.1)	45 (11.3)	33 (8.9)	
Highest level of education				0.044
Primary	196 (25.5)	114 (28.5)	82 (22.2)	
Secondary	331 (43.0)	173 (43.3)	158 (42.8)	
College or higher	242 (31.5)	113 (28.3)	129 (35.0)	
Occupation				0.655
Non-manual	55 (7.2)	32 (8.0)	23 (6.3)	
Manual, domestic service, agriculture	342 (44.8)	175 (44.0)	167 (45.8)	
Student	246 (32.2)	116 (29.1)	130 (35.6)	
Unemployed	120 (15.7)	75 (18.8)	45 (12.3)	
Marital status				0.060
Married	227 (29.7)	122 (30.7)	105 (28.6)	
With regular partner	414 (54.2)	204 (51.4)	210 (57.2)	
Single/no regular partner	91 (11.9)	50 (12.6)	41 (11.2)	
Divorced/separated	32 (4.2)	21 (5.3)	11 (3.0)	
Number of children				0.226
No children	330 (43.1)	163 (40.9)	167 (45.5)	
1–2 children	277 (36.2)	141 (35.3)	136 (37.1)	
3–4 children	108 (14.1)	67 (16.8)	41 (11.2)	
5+ children	51 (6.7)	28 (7.0)	23 (6.3)	
Fertility intentions				0.921
No children	215 (29.1)	119 (30.9)	96 (27.1)	
Less than 2 years	34 (4.6)	15 (3.9)	19 (5.4)	
More than 2 years	386 (52.2)	188 (48.8)	198 (55.9)	
After marriage	92 (12.4)	52 (13.5)	40 (11.3)	
Other	12 (1.6)	11 (2.9)	1 (0.3)	
Most effective method ever previously used				0.780
No method	51 (7.1)	24 (6.4)	27 (7.9)	
Long-acting reversible contraception	117 (16.3)	61 (16.2)	56 (16.3)	
Short-term method	540 (75.0)	288 (76.4)	252 (73.5)	
Rhythm method	12 (1.7)	4 (1.1)	8 (2.3)	
Using FP immediately prior to the abortion				0.469
No	528 (69.1)	270 (68.0)	258 (70.3)	
Yes	236 (30.9)	127 (32.0)	109 (29.7)	
Type of abortion				0.012
Medical	284 (37.1)	168 (42.2)	116 (31.5)	
Surgical	482 (62.9)	230 (57.8)	252 (68.5)	

*Notes*: *P*-value is for difference between baseline and post-intervention group

#### Sample characteristics

Over half the women interviewed were aged 18–24, and one third had completed college education or higher (Table 1). One third were currently studying, and nearly half were working in manual, domestic service or agricultural occupations. Most women were married (30%) or had a regular partner (54%). Less than 5% of women wanted a child within 2 years. Only 7% had never used a FP method, and 16% had used LARC previously. Client characteristics, including age, level of education, and marital status varied between clinics.

#### FP counselling received

Sixty-one percent of women reported that the provider counselled them on ways to prevent pregnancy at post-intervention, compared to 55% at baseline (*p* = 0.051) (Table 2). A greater proportion of women reported that the provider asked them which FP methods they had used before (71% vs. 58%, (*p* < 0.001) and whether they had experienced problems with these methods (47% vs. 37%, *p* < 0.001) at post-intervention. The mean number of methods women were counselled on increased from 2.9 to 3.3 (*p* = 0.002).

**Table 2:** Counselling received at post-intervention and baseline and satisfaction with overall service

	Baseline	Post-intervention	
Counselling received	*N* (%)	*N* (%)	*p*-value
Provider gave information about pregnancy prevention^a^	218 (54.5)	223 (61.4)	0.051
Provider asked about FP methods used before^a^	231 (57.8)	263 (71.3)	< 0.001
Provider asked about problems with previous methods^a^	146 (36.8)	174 (47.2)	< 0.001
Mean number of methods counselled on^c^	2.9	3.3	0.002
Reasons given for not taking same day PAFP at baseline and post-intervention, among those who did not receive a method			
Method not available or too expensive^b^	29 (9.0)	26 (10.2)	0.834
Partner was not available to give consent^b^	2 (0.6)	8 (3.1)	0.047
Family planning not needed^b^	13 (4.0)	17 (6.7)	0.182
Opposition to FP^b^	1 (0.3)	1 (0.4)	0.867
Doesn’t like FP^b^	25 (7.8)	38 (15.0)	0.011
Undecided^b^	207 (64.3)	131 (51.6)	0.013
Did not receive any or enough information^b^	35 (10.9)	11 (4.3)	0.005
Client satisfaction with service			
Provider gave clear instructions^a^	231 (57.9)	253 (68.8)	0.001
Provider made client feel comfortable^a^	357 (89.7)	340 (92.1)	0.271
Provider took enough time^a^	371 (93.0)	355 (96.7)	0.023
Friendliness and respect from staff^a^	347 (87.0)	319 (87.2)	0.966
Price charged for overall service^a^	210 (52.9)	207 (56.9)	0.297
Procedure^a^	264 (66.7)	257 (70.4)	0.266
Overall experience^a^	344 (86.6)	314 (86.5)	0.92
Would recommend a friend^a^	373 (95.6)	345 (97.5)	0.266

Notes:

For reasons given for not taking same day PAFP at baseline, percentages may not add up to 100 as multiple responses were possible

For ‘provider gave clear instructions’, ‘provider made client feel comfortable’, and ‘provider took enough time’, table shows percentage reporting this was true

For ‘staff were friendly and respectful’, ‘price charged for overall service’, ‘procedure’ and ‘overall experience’, table shows percentage reporting this as good or very good

For ‘would recommend a friend’, table shows percentage reporting they would be likely or very likely to recommend a friend

Denominators:

^a^All women

^b^ Women who did not receive a method

^c^Women who received counselling on pregnancy prevention

#### Reasons for not taking a FP method on the day of the procedure

Among women who did not take a FP method on the day of the abortion, the proportion reporting that it was because they did not receive enough information about FP declined from 11 to 4% (*p* = 0.005) The proportion reporting that it was because they were undecided declined from 64 to 52% (*p* = 0.013) (Table 2) between baseline and post-intervention.

#### Satisfaction with service

At both post-intervention and baseline, women reported high levels of satisfaction with all elements of the service except the cost (Table 2). More women reported that the provider gave clear instructions at post-intervention compared to baseline (69% vs. 58%, *p* = 0.001), and more reported that the provider took enough time to understand them (97% vs. 93%, *p* = 0.023).

#### Uptake of PAFP

Women were more likely to receive same-day PAFP at post-intervention than baseline (aOR 1.94, *p* < 0.001) (Table 3). Women were also more likely to leave with LARC on the same-day at post-intervention than baseline (aOR 1.72, *p* = 0.035). Method mix did not change between baseline and post-intervention (results not shown). There was some weak evidence that women who did not obtain a FP method on the same-day, and who were followed up, were less likely to obtain a method within 14 days of the abortion after the intervention compared to before (Table 3). There was no difference in the odds of receiving any PAFP within 14 days of the abortion in the post-intervention compared to the baseline group, among women that were followed up.

**Table 3:** Crude and adjusted odds of experiencing all family planning outcomes at post-intervention vs. baseline

	Denominator	*N* (%)	cOR and 95% CI	*P*-value	aOR and 95% CI	*P*-value
Received same day PAFP^a^						
Baseline	399	77 (19.30)	1.00	.	1.00	.
Post-intervention	367	113 (30.79)	2.13 (1.46–3.10)	< 0.001	1.94 (1.29–2.93)	0.001
Received same day LARC^a^						
Baseline	397	50 (12.59)	1.00	.	1.00	.
Post-intervention	365	71 (19.45)	1.92 (1.23–3.01)	0.004	1.72 (1.04–2.84)	0.035
Received PAFP 2–14 days post-abortion^b^						
Baseline	195	66 (33.85)	1.00	.	1.00	.
Post-intervention	124	29 (23.39)	0.60 (0.36–0.99)	0.048	0.57 (0.31–1.03)	0.064
Received any PAFP within 14 days of abortion^c^						
Baseline	238	109 (45.80)	1.00	.	1.00	.
Post-intervention	176	81 (46.02)	1.10 (0.72–1.67)	0.658	1.12 (0.70–1.80)	0.640

Notes:

Adjusted for age group, education, occupation, marital status, number of children, fertility intentions, type of abortion (surgical or medical), clinic, and use of family planning prior to the abortion

Denominators:

^a^All women

^b^Women who did not receive same day PAFP, who were followed up

^c^Women who were followed up

### Qualitative

When asked about how their provision had changed since the intervention started, some of the providers mentioned improved record keeping, increased PAFP provision, and general quality improvements, such as refreshed knowledge on the medical abortion regimen. One provider noted that, *“you know a human brain…*? *forgetting also is an issue, so being reminded through supervision or an induction or update is important”.*

The providers reported that the 1-day orientation was useful because they received new information, were able to speak to other providers, and felt encouraged. However, two of the providers desired more in-depth training, and many desired more regular training and updates. Several of the providers did not immediately remember the job aide, but a few providers reported using it occasionally and said that it helped them assess eligibility for FP methods.

Several providers noted that they had seen a change in the structure of their supervision visits and now regularly discussed quality issues including FP provision with their supervisor. Providers characterised the visits as providing encouragement and quality assurance: *“Well, they support us by encouraging us, where we have relaxed, they tell us not to relax and this makes the number bigger.”* A few of the providers stated that they felt their PAFP counselling and uptake had improved as a result of the visits, in part because of advice received on ways to improve uptake. The providers also noted their record keeping on abortion and contraception had improved as the supervisors looked through their records during each visit and advised on how to improve. However, one provider did not seem to have noticed any changes in their supervision visits, and spoke about unrelated activities, suggesting that for some, the structured supervisions may not have stood out as different from other existing activities.

Providers spoke about a range of barriers to uptake of PAFP. These included negative attitudes to contraception, for example, parents’ concerns about contraception causing promiscuity, concerns about side effects, misconceptions about the impact of contraception on long-term fertility and health, and religious beliefs. Other reasons given for not taking contraception included irregular sex, the woman deciding they will wait to have sex again until after marriage, wanting to complete the abortion process first, and the need for spousal consent. These barriers result in delays in decision-making, but some providers reported that clients who say they will return rarely do, because they have far to travel, they forget or they do not want to return to a facility where they have debt. Cost was also mentioned as a factor, as some cannot afford the added cost of contraception, or will go to government clinics or chemists for cheaper options.

## Discussion

The quality management intervention was associated with increased uptake of same-day PAFP, including increased uptake of LARC. Although there was no strong evidence of an increase in the *provision* of PAFP counselling following the intervention, the rise in same day PAFP uptake may reflect improvements in the *quality* of the counselling given, as providers were discussing previous use of contraception and problems experienced with them. Among women who did not receive a method, the proportion reporting that this was because they were undecided declined, suggesting that providers may have given them clearer information. While women were more likely to receive same-day PAFP following the intervention, uptake 2–14 days after the abortion did not increase. The findings suggest that the intervention may not have increased uptake of PAFP overall, but that it resulted in women obtaining it sooner after the abortion (i.e. those women who would have returned were provided with it on the day). However, as the follow up rates in this study were low, the results on uptake 2–14 days after, and within 14 days of, the abortion should be interpreted cautiously.

This intervention aimed to improve provider behaviour on PAFP counselling, and through this, increase PAFP uptake. Provider behaviour improved post-intervention, with higher quality counselling and higher satisfaction levels. The existing literature on the effect of contraceptive counselling on PAFP uptake is mixed [7, 10, 11]. A recent study, which included theory-based video as part of FP counselling, did not see any positive effect on LARC uptake in the intervention arm [12]. A separate study found that FP counselling incorporating motivational interviewing led to higher uptake of LARC, 3 month LARC continuation and patient satisfaction compared with ‘standard’ FP counselling [13]. In our study, although the improvement in provider behaviour may have reduced some of the barriers to same-day uptake of PAFP and helped women choose and obtain a method more quickly after their abortion, there was no evidence that it increased overall PAFP uptake in the 2 weeks following abortion. Nevertheless, an increase in same-day uptake means that more women are protected from unintended pregnancy sooner after the abortion, and do not face further financial and time-related costs to obtain a method during the period after the abortion.

The in-depth interviews suggest that the training and supervision visits may have been the most effective components of the intervention, in continuously reminding and encouraging providers to maintain quality. The job aide material did not seem to be commonly used. Previous research in sexual and reproductive health has also found supervision to be an important component of quality improvement [14, 15]. An overview of the evidence on strategies for improving health worker performance showed that, in general, the most effective strategies were those involving supervision, and that provision of written guidelines without additional interventions was generally ineffective [16]. It also found that multi-faceted interventions, which address more than one aspect of performance, might be more likely to improve health worker performance than individual interventions. Although this intervention had several components, a more comprehensive approach that specifically targeted more elements of performance may have been more effective in increasing uptake of PAFP.

### Recommendations for research and practice

As the intervention did not lead to an increase in the proportion of women obtaining PAFP 2–14 days post-abortion, more research into what, at a facility level, might reduce the barriers to obtaining PAFP later (e.g. reducing stock-outs, subsidising travel costs), would be helpful. Our qualitative findings provide some insight into the elements of quality management that are effective, and suggest that incorporating supportive supervision into practice may improve the quality of PAFP counselling. Further research examining in more detail the processes by which supportive supervision influences provider behaviour may support further improvements in quality management. Finally, even when provider and facility level barriers to provision of PAFP are removed, there are likely to be reasons why women choose not to take a family planning method. In-depth interviews with post-abortion women that took part in this study suggest that women may prefer not to make a decision on the day of the abortion procedure, because they want time to physically or emotionally recover from their abortion, or to consult with their partner (Penford-Taylor S, Wendot S, Nafula I, Footman K: A qualitative study of safe abortion and post-abortion family planning service experiences of women attending private facilities in Kenya, unpublished). A high proportion of women in our study stated that their reason for not taking FP on the day of their abortion was that they were undecided. Services must therefore find ways to support women after they leave the clinic, either through mobile technologies or in-person support.

### Limitations

The study design was limited by its single group design; too few clinics met the eligibility criteria to enable the inclusion of a comparison group in the study. It is not possible to know whether FP uptake would have increased anyway, in the absence of the intervention. However, the results suggest that quality of counselling and client satisfaction with elements of care relevant to the intervention were the only ones to have improved, which supports the notion that the increase in uptake was related to the intervention. The clinics were not randomised to the intervention, and there were differences in client characteristics between baseline and post-intervention groups. Although we adjusted the analyses for potential confounders, there may be unmeasured confounding, resulting in bias in our estimates. There may be a selection effect at the clinic level, whereby the clinics that took part in the study were those that were more motivated to improve quality and client satisfaction and committed to adhering to the quality management intervention. This would overestimate the association between PAFP uptake and the intervention. There is a risk of social desirability bias in the in-depth interviews with providers and we cannot rule out the possibility that providers may have changed their behaviour following the intervention simply because they were aware of the aims of the study. The response rate amongst women who were approached to take part in the study was high, but as research assistants were available to interview clients during working hours only, women who attended outside of these hours or on weekends were not approached. It is possible that our sample is not representative of all women accessing safe abortion and PAC services, for example, if women who visit outside of working hours differ in their likelihood of accepting PAFP. The follow up rate in this study was relatively low, and there were differences between women who were followed up and those who were not. There was no difference between women who were followed up and women who were not in same-day uptake of PAFP, but this does not necessarily mean that the women lost to follow up would not differ in their likelihood of obtaining PAFP 2–14 days after the abortion. If women who were not followed up were *more* likely to obtain PAFP 2–14 days after the abortion than women who were followed up, our results may underestimate the association (or mask a positive association) between the intervention and overall 2-week PAFP uptake. Conversely, if women who were not followed up were less likely to obtain PAFP 2–14 days after the abortion than women who were followed up, our results may overestimate the association (mask a negative association). Finally, to determine whether women had received FP counselling, they were asked if the provider had given them counselling or information about ways to prevent pregnancy. This may be interpreted quite broadly, and may explain why more women reported that the provider had asked them about previous FP method use than reported that the provider had given them information about pregnancy prevention.

## Conclusion

The results suggest that the intervention was successful in improving provider behaviour with regard to quality of FP counselling and provision or PAFP. Uptake of same-day PAFP, including LARC, did increase, but there was no overall increase in uptake of PAFP in the 2 weeks following the abortion. Interviews with providers suggest that supportive supervision was the most effective component of the intervention.

## Abbreviations

FP: Family planning
PAFP: Post-abortion family planning
LARC: Long acting reversible contraception
